# Diagnosis of Hypophosphatasia in Adults Presenting With Metatarsal Stress Fracture: Proof‐of‐Concept for a Case‐Finding Strategy

**DOI:** 10.1002/jbm4.10495

**Published:** 2021-04-02

**Authors:** Kenna Koehler, Said Atway, James Pipes, Steven Ing

**Affiliations:** ^1^ Ohio State University College of Medicine Columbus OH USA; ^2^ Division of Podiatry Ohio State University Wexner Medical Center Columbus OH USA; ^3^ Department of Internal Medicine, Division of Endocrinology, Diabetes and Metabolism Ohio State University Wexner Medical Center Columbus OH USA

**Keywords:** DISEASES AND DISORDERS OF BONE, DISORDERS OF CALCIUM/PHOSPHATE METABOLISM, INJURY/FRACTURE HEALING, MATRIX MINERALIZATION

## Abstract

Hypophosphatasia (HPP) is caused by loss‐of‐function mutations in *ALPL* resulting in decreased alkaline phosphatase (ALP) activity. Metatarsal stress fracture (MSF) is a common clinical feature of hypophosphatasia in adults. In this study, the primary objectives were to determine whether new cases of *ALPL* variants could be identified in patients with MSF and who also had serum ALP concentration below the reference range and to phenotype their clinical course. Electronic health records were queried for adult patients with MSF using International Classification of Disease codes (ICD‐9, ICD‐10CM) and ALP measurements. Patients with ALP levels below the normal limit were invited to receive mutational analysis of *ALPL* and to complete the following surveys: the Short Form 36 version 2 (SF36v2), the Brief Pain Inventory‐Short Form (BPI), and the Health Assessment Questionnaire Disability Index (HAQ‐DI). Cases with and controls without *ALPL* pathogenic variants were compared by survey scores and clinical variables relevant to fracture. In 1611 patients with MSF presenting to a podiatry clinic (10/1/2011–10/1/2017), 937 had ALP measurement, of whom 13 (1.4%) had ALP levels below the lower normal limit. In eight patients consenting to participate, two had heterozygous pathogenic *ALPL* variants. *ALPL* variants were found in 2 of 1611 patients (0.12%) with MSF, 2 patients of 937 (0.21%) in those with MSF and any ALP measurement, and 2 of 13 patients (15%) in MSF and decreased ALP level. Cases versus controls rated lower scores on eight of eight SF36v2 scales (range, 0–100); higher scores for worst pain (8.0 vs. 0.8) and average pain (6.0 vs. 0.7) on the BPI (range, 0–10); and higher standard disability score (1.4 vs. 0) on the HAQ‐DI (range, 0–3). These data provide proof‐of‐concept for HPP case identification in patients presenting to a podiatry clinic with MSF, suggesting a search for historically low ALP levels may be a useful step for consideration of HPP diagnosis, and supports a prospective study to determine an optimal case‐finding strategy. © 2021 The Authors. *JBMR Plus* published by Wiley Periodicals LLC on behalf of American Society for Bone and Mineral Research.

## Introduction

Hypophosphatasia (HPP), initially described by Rathbun in 1948,^(^ ^1^ ^)^ is a rare metabolic bone disorder characterized by loss‐of‐function mutations in the gene (*ALPL*) that encodes tissue nonspecific alkaline phosphatase.^(^ ^2^ ^)^ HPP is inherited in both an autosomal dominant and recessive manner with at least 411 reported mutations.^(^ ^3^ ^)^ Pathogenic *ALPL* mutations result in decreased activity of alkaline phosphatase (ALP), a critical enzyme for skeletal and dental mineralization.^(^ ^4^ ^)^ In HPP, alkaline phosphatase is deficient, leading to accumulation of extracellular inorganic pyrophosphate that inhibits hydroxyapatite nucleation and subsequently impairs bone mineralization.^(^ ^4^ ^)^ The traditional nosology of HPP classifies categories based on age of presentation of symptoms and signs: perinatal lethal, prenatal benign, infantile, childhood, adult, and odonto‐HPP.^(^ ^2^ ^)^ Accordingly, the clinical features of the disease exhibit a wide range of severity, from perinatal fatality (most severe) to odonto‐HPP (most mild).^(^ ^5^ ^)^ Toward the milder end of the spectrum, adults with HPP frequently present in middle age with nonspecific musculoskeletal complaints, such as bone pain and metatarsal stress fractures, which can be multiple and/or slow to heal.^(^ ^6^ ^)^ Low serum ALP concentrations, hypophosphatasemia, may be recognized retrospectively or newly discovered, and further history‐taking and medical record review may elucidate symptoms and signs consistent with HPP manifesting during childhood despite diagnosis in adulthood.^(^ ^7^, ^8^, ^9^ ^)^

Mornet and colleagues estimated the prevalence of mild to moderate forms of HPP (juvenile, adult, and odonto‐HPP) at 1 in 6379 individuals (0.016%) based on genetic modeling in a European population.^(^ ^10^ ^)^ In an electronic health record (EHR) query of more than 800,000 patients, McKiernan and colleagues showed persistently low ALP values in 1 of 1544 individuals (0.065%) and as a group exhibited more crystalline arthritis, chondrocalcinosis, calcific periarthritis, enthesopathy, and diffuse idiopathic skeletal hyperostosis than the general adult patient population: findings consistent with adults with HPP.^(^ ^11^ ^)^ Our primary goal was to determine how many cases of metatarsal stress fractures (MSF) and low ALP levels presenting to a podiatry clinic at our institution have HPP. Additionally, we sought to phenotype the clinical course and symptoms of these HPP patients compared with controls.

## Materials and Methods

### Study design and setting

**Fig. 1:**
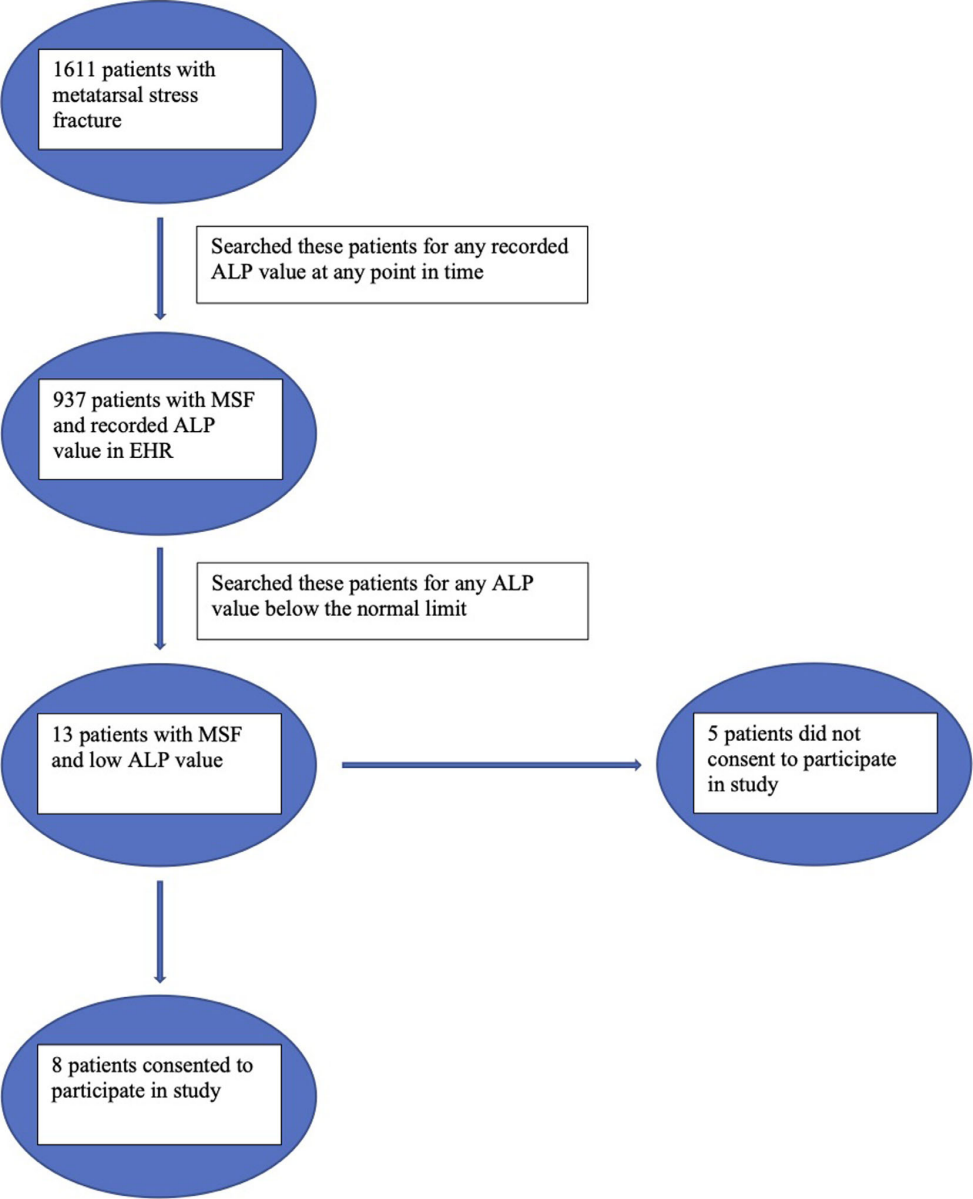
Flow chart illustrating study design. ALP indicates alkaline phosphatase; EHR, electronic health record; MSF, metatarsal stress fracture.

We conducted an institutional review board‐approved (OSU 2018H0331), single‐site, retrospective EHR review of patients ≥18 years of age with MSF presenting to a podiatry clinic and who had serum ALP measurement at the Ohio State University Wexner Medical Center (OSUWMC). We searched for initial podiatry clinic visits during a 6‐year period (10/1/2011‐10/1/2017) using *International Classification of Disease* diagnosis codes (*ICD‐9* and *ICD‐10CM*) for MSF (733.94, M84.376A). Metatarsal fracture (825.25, S02.309A) was also included in our search as these diagnosis codes may have been applied instead. The ALP reference range was defined as 38–126 U/L before and 32–126 U/L after June 24, 2013, the implementation date of a new assay platform (Beckman Coulter DxC to Beckman Coulter AU). Reference ranges were derived from sampling a local primary care practice population. Patients with MSF and low ALP levels were eligible to participate. We contacted eligible patients to invite participation and obtained informed consent (Fig. 1).

### Population

All patients who agreed to participate were invited to a single study visit during which blood was sampled for mutational analysis of *ALPL* (Prevention Genetics). Three groups are defined in this study: (i) Cases are patients with MSF, low ALP value, and pathogenic or likely pathogenic *ALPL* variant; (ii) control‐low ALP are patients with MSF, low ALP level, and negative *ALPL* mutation; and (iii) control‐normal ALP. For this last group, we matched five patients by age, sex, and race for each case. Control‐normal ALP patients had MSF, normal ALP level, were not contacted to participate in this study, and therefore did not have *ALPL* genetic mutation analysis or other assessments. Information technology staff searched the EHR and provided a single ALP measurement that we used to separate patients into these three groups. Because measurement of ALP is currently not a standard evaluation in the management of MSF, we imposed no limits that date of ALP be within a certain period relative to fracture. We searched the EHR to find and display all other ALP measurements. Antiresorptive agents (bisphosphonates and denosumab) were not excluded, as identification of patients with a pathogenic *ALPL* variant who had prior or current exposure would also be an important finding and allow for a discussion of potential harm.^(^ ^12^ ^)^

### Measurements

Next‐generation sequencing (NGS) technologies were used to cover the coding regions of the targeted genes plus 10 bases of noncoding DNA flanking each exon. The DNA was captured using Agilent clinical research exome hybridization probes and sequenced using Illumina's reversible dye terminator platform using 150 × 150 bp paired end reads with an average coverage of 220×. Copy number variants were detected from NGS data. All differences from the reference sequences were assigned one of five interpretations (pathogenic, likely pathogenic, variant of uncertain significance, likely benign, and benign).

We invited all eligible patients with a low ALP value to complete the following surveys: Brief Pain Inventory‐Short Form (BPI),^(^ ^13^ ^)^ Health Assessment Questionnaire Disability Index (HAQ‐DI),^(^ ^14^, ^15^ ^)^ and the Short Form 36 version 2 (SF36v2).^(^ ^16^, ^17^ ^)^ The BPI (range, 0–10) includes worst pain (at a single point in time), average pain, composite pain (a mean severity score of worst, average, least, and current pain scores), and pain interference (measurement of how much pain has interfered with seven activities of daily living, including general activity, walking, work, mood, enjoyment of life, relations with others, and sleep) scores.^(^ ^13^ ^)^ The HAQ‐DI (range, 0–3) includes a standard disability index (disability level of the individual), alternative disability index (disability level of the individual when using aids and devices to compensate for disability), and pain scale (presence or absence of arthritis‐related pain and its severity) scores.^(^ ^14^, ^15^ ^)^ The SF36v2 (range, 0–100) includes eight scale scores (physical functioning, role–physical, bodily pain, general health, vitality, social functioning, role–emotional, and mental health) and two summary scores, the physical and mental component scores.^(^ ^16^, ^17^ ^)^ The first four and last four scales comprise physical and mental component scores, respectively.^(^ ^16^, ^17^ ^)^

The EHR was reviewed for demographic and clinical variables related to the metatarsal fracture and compared with matched controls. We specifically sought to find examples of multiple metatarsal fractures in which more than one metatarsal fracture presented in the same episode, other metatarsal fractures in which a fracture of the same or different metatarsal occurred during a separate episode, a history of nonmetatarsal fractures, delay in healing of metatarsal fracture (defined as delay in fracture healing at 6 months after fracture), nonunion of metatarsal fracture (fracture nonunion at 9 months after fracture), and metatarsal fractures requiring surgical fixation.

### Data analysis

All data are presented descriptively only because of the small sample size.

## Results

**Fig. 2:**
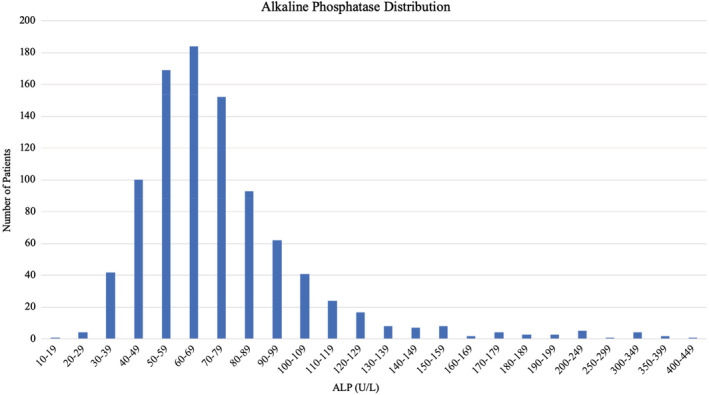
Distribution of serum alkaline phosphatase (ALP) concentration (U/L) among patients with metatarsal fracture.

A total of 1611 patients with MSF were identified from the EHR, of whom 937 had a recorded ALP measurement. Serum ALP values were normally distributed with a rightward skew (Fig. 2), range, 18–444 U/L. Most values (98%) fell within 30–199 U/L. There were 13 patients with low ALP value (1.4%). Of the 13 hypophosphatasemic patients (12 female, 1 male), eight consented to participate. Reasons for nonconsent in the other five patients included living out of state (two patients), unable to be reached (two patients), and not interested in participating (one patient). The ALP values that identified the patients for eligibility into the study in case and control‐low ALP groups are given in Table 1. All other ALP values available in the EHR are also provided. The date of ALP measurement compared with the date of fracture is also given in Table 1. ALP was drawn before fracture in five patients; there was one patient that had an ALP drawn at the same time as initial fracture presentation.

**Table 1:** ALP Values for Case and Control‐Low ALP Groups

Patient	Date of fracture	ALP (U/L)	Date of ALP	All other ALP values (U/L)
Cases				
1	08/2013	30	07/2005	25, 28, 33, 38, 48
2	09/2012	35	04/2003	42
Control‐low ALP				
1	05/2012	25	05/2012	
2	02/2015	35	01/2007	63, 69, 69, 94
3	03/2014	36	01/2004	47, 51, 53, 63
4	11/2012	23	04/2008	20, 24, 25, 26, 28, 29, 30, 31, 32, 36, 39
5	05/2013	25	02/2016	21, 22, 31
6	12/2011	31	01/2016	31, 33, 36, 41, 49

Abbreviation: ALP, alkaline phosphatase.

Note: Date of fracture is the date of metatarsal fracture; ALP (U/L) are the abnormal ALP values for cases and control‐low ALP groups that were captured for eligibility in study; Date of ALP is the date that the abnormal ALP was drawn compared with the date of fracture; All other ALP Values are all other recorded ALP values that were found in the electronic health record at any other point in time. The ALP reference range was defined as 38–126 U/L before and 32–126 U/L after June 24, 2013, the implementation date of a new assay platform (Beckman Coulter DxC to Beckman Coulter AU).

Demographic characteristics are given in Table 2. Mean ages were 54.0–61.5 years. Mean ALP value in control‐normal ALP group was 69.60 U/L, higher than the other two groups, as expected. All patients were White and female.

**Table 2:** Patient Demographics and Fracture Characteristics

Variable	Case	Control‐low ALP	Control‐normal ALP
No.	2	6	10
Age, mean, y	61.5	54.0	61.4
Gender, %, female (no.)	100 (2)	100 (6)	100 (10)
Race/ethnicity, %, White (no.)	100 (2)	100 (6)	100 (10)
ALP, U/L	32.50	29.17	69.90
Multiple MT fractures, % (no.)	50 (1)	17 (1)	0 (0)
Other MT fractures, % (no.)	50 (1)	33 (2)	10 (1)
Non‐MT fractures, % (no.)	100 (2)	50 (3)	40 (4)
Delay, % (no.)	0 (0)	0 (0)	0 (0)
Nonunion, % (no.)	50 (1)	0 (0)	10 (1)
Surgery, % (no.)	0 (0)	33 (2)	10 (1)

Abbreviations: ALP, alkaline phosphatase; MT, metatarsal.

Note: Demographic variables, ALP values, number of multiple metatarsal (>1 metatarsal fracture presented in same episode), number of other metatarsal fractures (fracture of the same or different metatarsal occurred during a separate episode), number of nonmetatarsal fractures sustained, history of delay in healing (defined as delay in fracture healing at 6 months after fracture), nonunion of fracture (fracture nonunion at 9 months after fracture), and requirement of surgical fixation for metatarsal fractures. Case is defined by MSF, low ALP, and pathogenic or likely pathogenic *ALPL* variant; control‐low ALP is defined by MSF, low ALP, and negative ALPL mutation; control‐normal ALP is defined by MSF, normal ALP, matched to cases 5:1 by age, sex, and race.

In eight patients with low ALP, two showed *ALPL* variants (cases). Mutations in both cases were heterozygous, autosomal dominant, and previously reported in more than one patient. One case patient harbored a pathogenic mutation and the other case patient had a likely pathogenic mutation (Table 3). The other six patients showed no evidence of mutation, including variants of uncertain significance. Copy number variants were not found within the genomic region encompassing the *ALPL* gene.

**Table 3:** Gene Mutation Characteristics

DNA variations, predicted effects, zygosity	Mode of inheritance	Interpretation	Variant previously reported
c.318 G > C, p.Gln106His, heterozygous	Autosomal Dominant	Likely Pathogenic	1 patient with perinatal HPP; 3‐generation family with autosomal dominant HPP
c.407 G > A, p.Arg136His, Heterozygous	Autosomal Dominant	Pathogenic	1 patient with infantile HPP (compound heterozygous); 1 patient with odonto‐HPP

Abbreviations: AD, autosomal dominant; HPP, hypophosphatasia.

Note: Gene mutation characterization for the two positive mutations in the study; DNA variation, predicted change in protein structure, zygosity, inheritance pattern, pathogenicity (benign, likely benign, variant of uncertain significance, likely pathogenic, pathogenic), and variant information from the database.^(^ ^3^ ^)^

In the two patients with *ALPL* variants, one patient presented with multiple metatarsal fractures and had another MT fracture before index fractures. Both patients also had a history of nonmetatarsal fracture (Table 2). The first patient recalls a clavicular fracture in childhood from a standing‐height fall, an episode of right foot pain and radiographic finding of right‐sided metatarsal fracture two decades before index fractures; both prior fractures healed without complications. She presented with right fourth and fifth metatarsal stress fractures. The second patient had traumatic nondisplaced radial head fracture before index fracture and proximal fifth toe phalanx fracture after index fracture; both healed without complications. There were four nonmetatarsal fractures among three patients in the control‐low ALP group: one with a femur and tibial stress fracture, another with a humeral head fracture, and the third with a radial head fracture. Details of fragility mechanisms of these fractures could not be adequately gleaned via chart review (and per protocol there was no further contact with these patients).

One case patient experienced fracture nonunion compared with none in the control‐low ALP and one in the control‐normal ALP groups (0% and 10%). Two patients in the control‐low ALP and one in the control‐normal ALP group required surgical fixation of their fractures (17% and 10%, respectively) versus none in the case group.

**Fig. 3:**
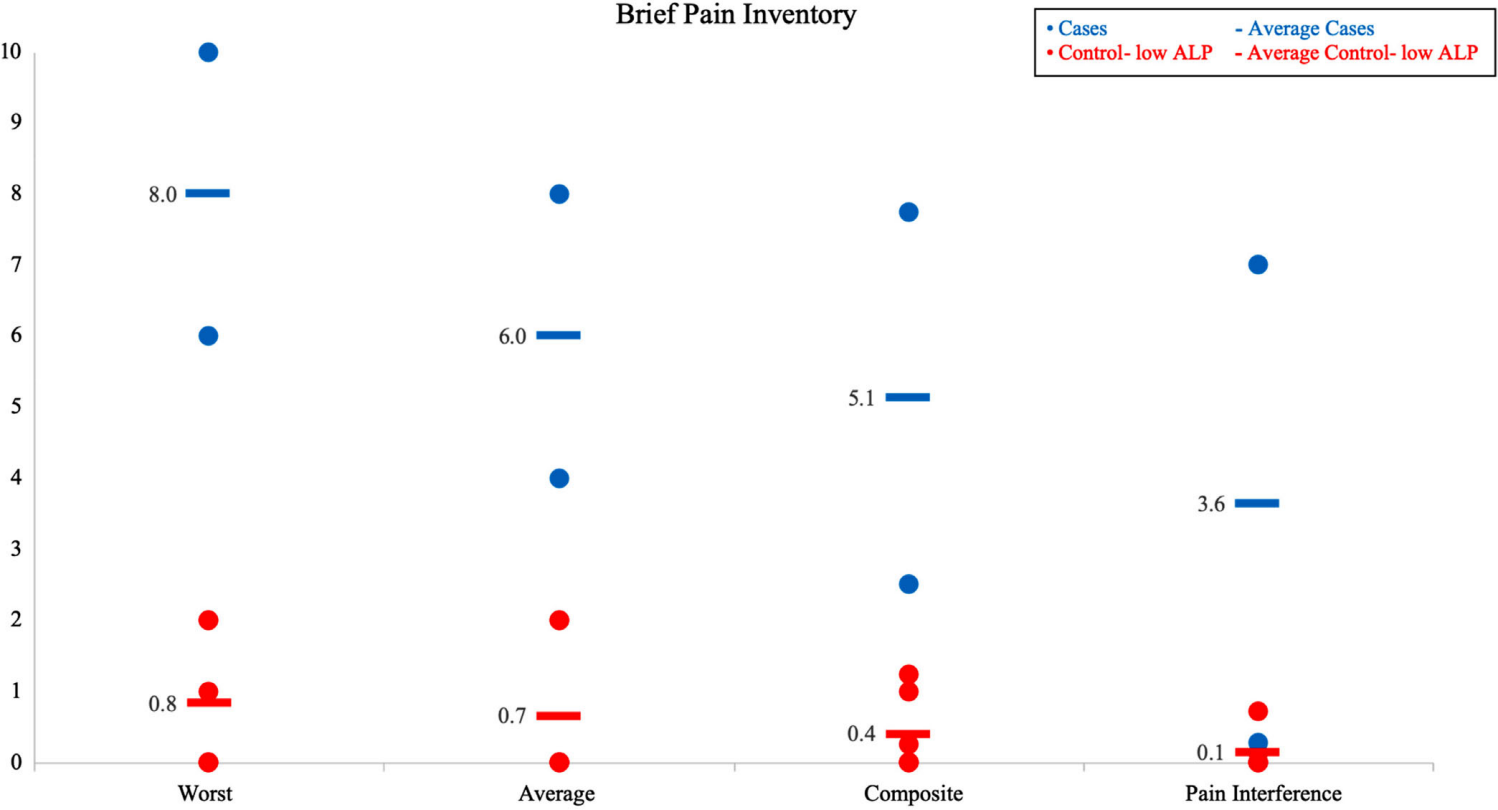
Brief pain inventory; range, 0–10. ALP indicates alkaline phosphatase.

**Fig. 4:**
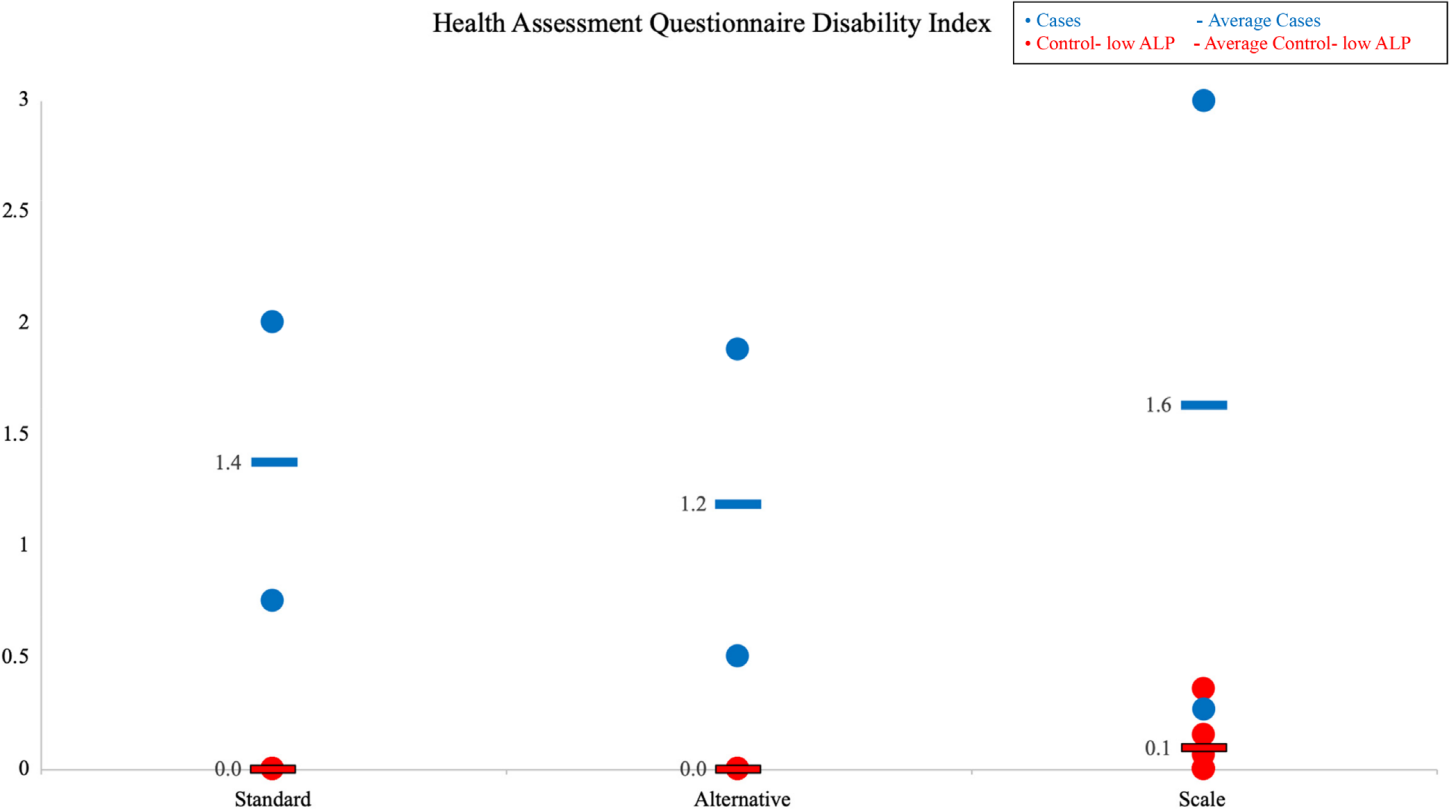
Health assessment disability index; range, 0–3. ALP indicates alkaline phosphatase.

**Fig. 5:**
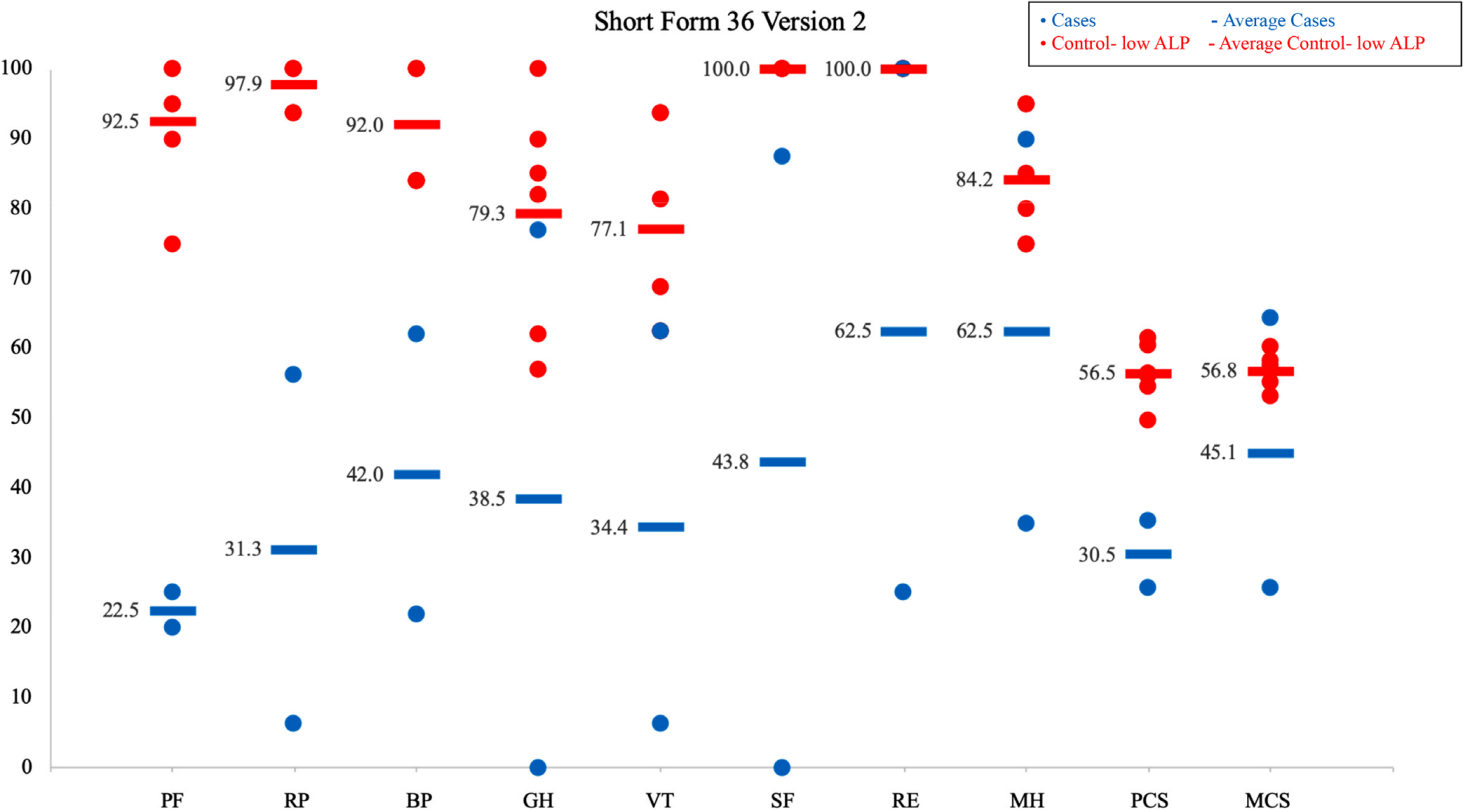
Short form 36 version 2; range, 0–100. ALP indicates alkaline phosphatase; BP, bodily pain; GH, general health; MCS, mental component score; MH, mental health; PCS, physical component score; PF, physical functioning; RE, role–emotional; RP, role–physical; SF, social functioning; VT, vitality.

Cases had higher scores for worst, average, composite, and pain interference scores (8.0, 6.0, 5.1, and 3.6, respectively) than control‐low ALP patients (0.8, 0.7, 0.4, and 0.1, respectively; Fig. 3). Cases had higher scores for the standard disability index, the alternative disability index, and the pain scale (1.4, 1.2, and 1.6, respectively) compared with control‐low ALP patients (0, 0, and 0.1, respectively; Fig. 4). Cases versus controls also had worse scores on eight out of eight scales and worse physical component (30.5 vs. 56.5) and mental component scores (45.1 vs. 56.8; Fig. 5).

Upon chart review, both cases showed radiographic evidence for metatarsal fracture and were subsequently treated with a walking boot. Both cases also showed childhood symptoms consistent with HPP. In case 1, chronic lower extremity pain attributed to “growing pains” limited her ability to participate in physical activities and contributed to recurrent falls and difficulty “keeping up” with her childhood peers because of fatigue. The patient in case 2 endorsed chronic fatigue and muscle weakness during childhood. In addition, both cases had poor dentition and by their early 20s had full upper extraction (case 1) or multiple crowns/root canals (case 2).

## Discussion

In this pilot study, we aimed to determine how many cases of MSF and low ALP values who presented to a podiatry clinic harbored a pathogenic or likely pathogenic *ALPL* variant. The prevalence of a pathogenic *ALPL* variant in a population of MSF was 0.12% (2 of 1611); MSF and recorded ALP measurement was 0.21% (2 of 937); and MSF and low ALP measurement was 15% (2 of 13). Compared with the Mornet and colleagues general population‐prevalence estimate for mild to moderate forms of HPP (0.016%), this population with MSF and MSF plus hypophosphatasemia are roughly one and three orders of magnitude greater, respectively.^(^ ^10^ ^)^ These data support the concept that case‐finding of HPP is enriched when performed in a population with HPP clinical manifestations. Rauch and colleagues examined a cohort of patients with hypophosphatasemia and rheumatologic symptoms in an adult metabolic bone clinic and performed *ALPL* sequencing.^(^ ^18^ ^)^ Heterozygous pathogenic or likely pathogenic *ALPL* variants were identified in 14 of 24 (58%) patients.^(^ ^18^ ^)^ The present study adds to the case‐finding strategy by HPP clinical manifestations and low ALP levels.

Cases were more likely to have a history of nonmetatarsal fractures, other metatarsal fractures, and multiple metatarsal fractures compared with patients without the mutation. Describing the disease burden in adults with HPP, Seefried and colleagues found that 17.9% experienced three or more fractures or pseudofractures in their lifetime.^(^ ^19^ ^)^ Similarly, cases in the present study showed a pattern of multiple (≥2) metatarsal fractures and at least one other nonmetatarsal fracture. Cases had worse physical component and mental component scores, pain score, and disability score versus controls with negative mutation in the present study, with similar patterns to this HPP patient registry.^(^ ^19^ ^)^ Whereas our cases were comparable with the HPP Global Registry in pain interference (3.6 vs. 3.3) and SF36v2 mental component scores (45.1 vs. 45.0), cases rated worse average pain (6.0 vs. 3.5), standard disability (1.4 vs. 0.5), and SF36v2 physical component scores (30.5 vs. 41.7) in our study.^(^ ^19^ ^)^

The mutation in case 1 (c.318G > C; p.Gln106His) was previously reported in two neonates presenting with perinatal HPP and neonatal epileptic encephalopathy, as well as another *ALPL* variant.^(^ ^20^, ^21^ ^)^ The mutation in case 2 (c.407G > A, pArg136His) was reported in one patient with infantile HPP and another with odonto‐HPP.^(^ ^22^, ^23^ ^)^

One case patient showed nonunion at 9 months and two control‐low ALP patients eventually required surgical fixation for metatarsal fracture. Use of PTH 1–34 or 1–84 has been reported to treat nonhealing fractures in adults with HPP and could be considered in the event of future fractures that display delayed healing or nonunion.^(^ ^24^, ^25^, ^26^ ^)^ In the United States, enzyme replacement therapy is approved for adults with documented pediatric‐onset HPP and is not indicated for episodic use postfracture.

ALP levels increase following fracture corresponding to fracture healing in the general population. In a cohort of patients with traumatic fracture of tibia–fibula (not selected for diagnosis of HPP), ALP levels on the day of fracture were normal, increased by week 2, and peaked at 3 weeks postfracture before returning to normal at the completion of healing.^(^ ^27^ ^)^ Compared with patients who healed at a normal time (by 6 months), those with delayed healing at 6 months and nonunion at 9 months showed blunted ALP response; however, the delayed healing and nonunion groups still showed higher ALP levels versus baseline (1.5 and 2.5‐fold elevations, respectively).^(^ ^27^ ^)^ We are unaware of data on trajectory of ALP levels postfracture in HPP patients. ALP levels do increase in HPP patients who sustain a fracture and are treated with teriparatide or PTH (1–84).^(^ ^24^, ^25^, ^26^ ^)^ Nevertheless, an ALP rise postfracture remains a possibility; hence, measurement of ALP during the period of fracture healing could have misidentified patients with higher than baseline ALP (i.e., false negatives). Taking this into account, our study may have missed cases; thus, data derived is a conservative estimate of the proportion of patients who could be identified based on low ALP levels. An optimal case‐finding strategy may need to sample ALP levels at such a time that avoids possible confounding by fracture healing.

There are alternative causes of hypophosphatasemia other than HPP, including cardiac bypass surgery, celiac disease, clofibrate therapy, cleidocranial dysplasia, Cushing syndrome, severe hypothyroidism, improperly collected blood (EDTA), massive transfusion, milk–alkali syndrome, multiple myeloma, pernicious or profound anemia, radioactive heavy metals, starvation, vitamin C deficiency, vitamin D intoxication, Wilson disease, zinc or magnesium deficiency, and bisphosphonate use.^(^ ^2^, ^28^ ^)^ Comprehensive EHR review of our control‐low ALP patients revealed that one control patient had a history of anemia and another had a history of anemia and hypothyroidism, but neither were severe, and therefore an unlikely explanation for those low ALP values.

Antiresorptive therapy may lower ALP levels and contribute to false positives. On chart review, neither patient with *ALPL* variant (cases) had prior antiresorptive exposure. However, four out of six control‐low ALP patients did have a history of alendronate (ALN) use (patients #3–6 in Table 1). Thus, we attempted to determine the timing of ALN in relation to ALP measurement, and found that in two patients (#5 and #6), low ALP occurred while on ALN therapy. However, in the other two patients (#3 and #4), we could not determine that ALP was measured while taking this medication. Details on ALN use was unclear because (i) the ALN prescription was before implementation of EHR in our institution, and (ii) the prescriber was outside our network. Before performing a costly genetic test of *ALPL* in a patient with low ALP presenting with MSF, we suggest clinicians first review their history of antiresorptive therapy as a potential explanation for the low ALP level. In addition, circulating levels of pyridoxal‐5′‐phosphate (PLP) may be elevated patients with HPP.^(^ ^29^ ^)^ As such, clinicians should consider measuring PLP concentrations in patients presenting with MSF and low ALP as a low‐cost confirmatory test.

The identification of adults with HPP is clinically beneficial given the burden of disease and availability of emerging treatment options with enzyme replacement therapy. Additionally, these patients might be mistakenly diagnosed with osteoporosis or osteopenia and treated with antiresorptive medications, which should be avoided in this population. This case finding approach may help to avoid the inappropriate use of such medications and atypical femoral fractures.^(^ ^12^ ^)^

A limitation of this study was the small sample size, which prevented hypothesis testing and statistical analysis. Additionally, five patients with low ALP measurement did not participate in the study, which could have affected the number of positive *ALPL* mutations in our sample. Finally, one of the two cases seemed to report especially poor survey results. Given the small sample, the generalizability of these scores is unclear.

In summary, to our knowledge, this is the first report of a case‐finding strategy involving metatarsal fracture and prior ALP values. We recommend that podiatrists and other providers managing metatarsal fractures review the patient medical record for prior ALP levels as a no‐cost, simple screening tool to identify patients who could have undiagnosed HPP. This study provides preliminary data for a prospective study to evaluate such a case‐finding strategy in patients with metatarsal fracture to diagnose HPP.

## Author Contributions

**Kenna Koehler:** Formal analysis; investigation; writing‐original draft; writing‐review & editing. **Said Atway:** Data curation; writing‐review & editing. **James Pipes:** Data curation; writing‐review & editing. **Steven Ing:** Conceptualization; data curation; formal analysis; funding acquisition; investigation; methodology; project administration; writing‐original draft; writing‐review & editing. Study conduct: SI. Data collection: SI. Data analysis: KK and SI. Data interpretation: KK, SA, JP, and SI. Drafting manuscript: KK and SI. Revising manuscript content: KK, SA, JP, and SI. Approving final version of manuscript: KK and SI. KK takes responsibility for the integrity of the data analysis.

## Conflict of Interest

This study received funding and other support from Alexion Pharmaceuticals, Inc. SWI has received compensation for participation in Ad Hoc Advisory Board sponsored by Alexion Pharmaceutical.

### Peer Review

The peer review history for this article is available at https://publons.com/publon/10.1002/jbm4.10495.

## References

[1] Rathbun JC . Hypophosphatasia: a new developmental anomaly. Am J Dis Child. 1948;75(6):822‐831. 18110134 10.1001/archpedi.1948.02030020840003

[2] Whyte MP . Hypophosphatasia—aetiology, nosology, pathogenesis, diagnosis and treatment. Nat Rev Endocrinol. 2016;12(4):233. 26893260 10.1038/nrendo.2016.14

[3] http://www.sesep.uvsq.fr/03_hypo_mutations.php. [7 August 2020].

[4] Whyte MP . Physiological role of alkaline phosphatase explored in hypophosphatasia. Ann N Y Acad Sci. 2010;1192(1):190‐200. 20392236 10.1111/j.1749-6632.2010.05387.x

[5] Berkseth KE , Tebben PJ , Drake MT , Hefferan TE , Jewison DE , Wermers RA . Clinical spectrum of hypophosphatasia diagnosed in adults. Bone. 2013;54(1):21‐27. 23352924 10.1016/j.bone.2013.01.024

[6] Briot K , Roux C . Adult hypophosphatasia. Curr Opin Rheumatol. 2016;28(4):448‐451. 26962706 10.1097/BOR.0000000000000286

[7] Khandwala H , Mumm S , Whyte M . Low serum alkaline phosphatase activity and pathologic fracture: case report and brief review of hypophosphatasia diagnosed in adulthood. Endocr Pract. 2006;12(6):676‐681. 17229666 10.4158/EP.12.6.676

[8] Braunstein NA . Multiple fractures, pain, and severe disability in a patient with adult‐onset hypophosphatasia. Bone Rep. 2016;4:1‐4. 28326335 10.1016/j.bonr.2015.10.005 PMC4926841

[9] Belkhouribchia J , Bravenboer B , Meuwissen M , Velkeniers B . Osteomalacia with low alkaline phosphatase: a not so rare condition with important consequences. BMJ Case Rep. 2016;2016:bcr2015212827. 10.1136/bcr-2015-212827 PMC4735159 26823351

[10] Mornet E , Yvard A , Taillandier A , Fauvert D , Simon‐Bouy B . A molecular‐based estimation of the prevalence of hypophosphatasia in the European population. Ann Hum Genet. 2011;75(3):439‐445. 21488855 10.1111/j.1469-1809.2011.00642.x

[11] McKiernan FE , Berg RL , Fuehrer J . Clinical and radiographic findings in adults with persistent hypophosphatasemia. J Bone Miner Res. 2014;29(7):1651‐1660. 24443354 10.1002/jbmr.2178

[12] Sutton RA , Mumm S , Coburn SP , Ericson KL , Whyte MP . “Atypical femoral fractures” during bisphosphonate exposure in adult hypophosphatasia. J Bone Miner Res. 2012;27(5):987‐994. 22322541 10.1002/jbmr.1565

[13] Cleeland CS . The Brief Pain Inventory User Guide. The University of Texas MD Anderson Cancer Center; 2009.

[14] Wolfe F. A brief clinical health assessment instrument: CLINHAQ. Arthritis Rheum. 1989;32(suppl):S9.

[15] Ramey D , Fries J , Singh G . Quality of life and pharmacoleconomics in clinical trials, the health assessment questionnaire 1995‐status and review. In Spilker B , ed. Quality of Life and Pharmacoeconomics in Clinical Trials. 2nd ed. Lippincott‐Raven Publishers; 1996 pp 227‐237.

[16] Ware ME , Kosinski M , Dewey JE . How to Score Version 2 of the SF‐36 Health Survey (Standard & Acute Forms). Quality Metric Inc; 2001.

[17] Ware JE , Kosinski M , Turner‐Bowker DM , Gandeck B . User's Manual for the SF‐12v2TM Health Survey (with a Supplement Documenting SF‐12 Health Survey). Quality Metric Inc; 2007.

[18] Rauch F , Bardai G , Rockman‐Greenberg C . ALPL mutations in adults with rheumatologic disorders and low serum alkaline phosphatase activity. J Bone Miner Metab. 2019;37(5):893‐899. 30719581 10.1007/s00774-019-00991-4

[19] Seefried L , Dahir K , Petryk A , et al. Burden of illness in adults with hypophosphatasia: data from the global hypophosphatasia patient registry. J Bone Miner Res. 2020;35(11):2171‐2178. 32654183 10.1002/jbmr.4130

[20] Balasubramaniam S , Bowling F , Carpenter K , et al. Perinatal hypophosphatasia presenting as neonatal epileptic encephalopathy with abnormal neurotransmitter metabolism secondary to reduced co‐factor pyridoxal‐5′‐phosphate availability. J Inherit Metab Dis. 2010;33:25‐33. 20049532 10.1007/s10545-009-9012-y

[21] Taillandier A , Lia‐Baldini AS , Mouchard M , et al. Twelve novel mutations in the tissue‐nonspecific alkaline phosphatase gene (ALPL) in patients with various forms of hypophosphatasia. Hum Mutat. 2001;18(1):83‐84. 10.1002/humu.1154 11438998

[22] Mumm S , Jones J , Finnegan P , Henthorn PS , Podgornik MN , Whyte MP . Denaturing gradient gel electrophoresis analysis of the tissue nonspecific alkaline phosphatase isoenzyme gene in hypophosphatasia. Mol Genet Metab. 2002;75(2):143‐153. 11855933 10.1006/mgme.2001.3283

[23] Fauvert D , Brun‐Heath I , Lia‐Baldini AS , et al. Mild forms of hypophosphatasia mostly result from dominant negative effect of severe alleles or from compound heterozygosity for severe and moderate alleles. BMC Med Genet. 2009;10(1):51. 19500388 10.1186/1471-2350-10-51 PMC2702372

[24] Whyte MP , Mumm S , Deal C . Adult hypophosphatasia treated with teriparatide. J Clin Endocrinol Metabol. 2007;92(4):1203‐1208. 10.1210/jc.2006-1902 17213282

[25] Gagnon C , Sims NA , Mumm S , et al. Lack of sustained response to teriparatide in a patient with adult hypophosphatasia. J Clin Endocrinol Metabol. 2010;95(3):1007‐1012. 10.1210/jc.2009-1965 20089612

[26] Schalin‐Jäntti C , Mornet E , Lamminen A , Välimäki MJ . Parathyroid hormone treatment improves pain and fracture healing in adult hypophosphatasia. J Clin Endocrinol Metabol. 2010;95(12):5174‐5179. 10.1210/jc.2010-1168 20739387

[27] Ajai S , Sabir A , Mahdi AA , Srivastava RN . Evaluation of serum alkaline phosphatase as a biomarker of healing process progression of simple diaphyseal fractures in adult patients. Int Res J Biol Sci. 2013;2:40‐43.

[28] Riancho‐Zarrabeitia L , García‐Unzueta M , Tenorio JA , et al. Clinical, biochemical and genetic spectrum of low alkaline phosphatase levels in adults. Eur J Intern Med. 2016;29:40‐45. 26783040 10.1016/j.ejim.2015.12.019

[29] Whyte MP , Mahuren JD , Vrabel LA , Coburn SP . Markedly increased circulating pyridoxal‐5′‐phosphate levels in hypophosphatasia. Alkaline phosphatase acts in vitamin B6 metabolism. J Clin Invest. 1985;76(2):752‐756. 4031070 10.1172/JCI112031 PMC423894

